# The application of ozonated water rearranges the *Vitis vinifera* L. leaf and berry transcriptomes eliciting defence and antioxidant responses

**DOI:** 10.1038/s41598-021-87542-y

**Published:** 2021-04-14

**Authors:** Ana Campayo, Stefania Savoi, Charles Romieu, Alberto José López-Jiménez, Kortes Serrano de la Hoz, M. Rosario Salinas, Laurent Torregrosa, Gonzalo L. Alonso

**Affiliations:** 1 Cátedra de Química Agrícola, E.T.S.I. Agrónomos y de Montes, Universidad de Castilla-La Mancha, Avda. de España s/n, 02071 Albacete, Spain; 2 BetterRID (Better Research, Innovation and Development, S.L.), Carretera de Las Peñas (CM-3203), Km 3.2, Campo de Prácticas-UCLM, 02071 Albacete, Spain; 3 AGAP, CIRAD, INRAe, Institut Agro-Montpellier SupAgro, Montpellier University, 34060 Montpellier, France; 4 Departamento de Ciencia y Tecnología Agroforestal y Genética, Universidad de Castilla-La Mancha, Campus Universitario s/n, 02071 Albacete, Spain

**Keywords:** Transcriptomics, Abiotic, Plant physiology

## Abstract

Ozonated water has become an innovative, environmentally friendly tool for controlling the development of fungal diseases in the vineyard or during grape postharvest conservation. However, little information is currently available on the effects of ozonated water sprayings on the grapevine physiology and metabolism. Using the microvine model, we studied the transcriptomic response of leaf and fruit organs to this treatment. The response to ozone was observed to be organ and developmental stage-dependent, with a decrease of the number of DEGs (differentially expressed genes) in the fruit from the onset of ripening to later stages. The most highly up-regulated gene families were heat-shock proteins and chaperones. Other up-regulated genes were involved in oxidative stress homeostasis such as those of the ascorbate–glutathione cycle and glutathione S-transferases. In contrast, genes related to cell wall development and secondary metabolites (carotenoids, terpenoids, phenylpropanoids / flavonoids) were generally down-regulated after ozone treatment, mainly in the early stage of fruit ripening. This down-regulation may indicate a possible carbon competition favouring the re-establishment and maintenance of the redox homeostasis rather than the synthesis of secondary metabolites at the beginning of ripening, the most ozone responsive developmental stage.

## Introduction

*Vitis vinifera* encompasses most grapevine cultivars used for table grape and wine production. Unfortunately, this species is highly susceptible to a range of fungal diseases such as downy and powdery mildews and the grey mould, respectively caused by *Plasmopara viticola*, *Erysiphe necator* and *Botrytis cinerea*. Moreover, a complex group of pathogenic fungi that attacks perennial organs is responsible for the so-called grapevine trunk diseases. To overcome the negative impacts of these pathogens on plant development and fruit quality, and avoid excessive crop losses, viticulture needs to perform intense fungicide spraying programs, especially in hot and wet weather conditions. Even organic and biodynamic approaches largely require sulfur- and copper-based formulations that may be detrimental to the soil ecosystem in the long term. The ecological and environmental sustainability is an increasing concern for consumers and more generally for society.

One way to reduce the susceptibility of *V. vinifera* to pathogens is to breed new cultivars introgressing genetic traits of resistance from American and Asian *Vitis* spp. Several breeding programs are ongoing in Europe and abroad with an increment of new resistant genotypes available. In parallel to introducing new varieties, which is a long process and often not entirely accepted by the market, other strategies like the application of bioactive natural-derived products (silicons, laminarin, potassium phosphonates, analog of salicylic acid, phytomelatonin, etc.) that act as elicitors of plant biotic stress resistance^1,2^, or the use of ozone (O_3_) have been proposed as smart approaches to control fungal diseases. Indeed, when applied in aqueous solution, ozone has been shown to suppress spore germination of the esca-associated fungus *Phaeoacremonium aleophilum* and reduce fungal development by 50% on Cabernet Sauvignon cuttings^3^. The use of ozonated water in integrated vineyard pest management appears to be as effective as traditional chemical treatments in reducing fungal populations on leaves and grape bunches^4^. The efficiency of ozone is thought to lie in its oxidising potential, which translates into the ability to react with numerous cellular constituents hence a broad-spectrum antimicrobial action^5^.

Its low persistence after application makes ozone particularly attractive from an environmental point of view. This triatomic molecule is highly unstable and spontaneously decomposes into oxygen without leaving hazardous residues, with a shorter half-life in water than in the gaseous state^5^. In aqueous solution, ozone can be broken down via a chain reaction mechanism resulting in the production of reactive oxygen species (ROS), i.e. the hydroperoxide (HO_2_^·^), superoxide (^·^O_2_^−^) and hydroxyl (^·^OH) radicals and hydrogen peroxide (H_2_O_2_), all contributing to the high oxidising power of ozone^6^.

Ozone enters plant tissues through the stomata, lenticels or physical breaks in the cuticle. Then it reacts with molecules present in the apoplastic fluid, cell wall and plasma membranes, where it decomposes to produce the ROS mentioned above^7^. Under the oxidative stress induced by ozone and derived products, plants develop defence mechanisms at the genetic, transcriptional and biochemical level, which includes the synthesis of antioxidants such as ascorbate, glutathione, enzymes like superoxide dismutases, catalases and peroxidases, and secondary metabolites like carotenoids, terpenoids and phenolics^8–10^. When the detoxification capacity of plant cells is overwhelmed, cellular damage can occur.

Most research about the effects of ozone on plants has focused on the physiological changes triggered by ozone as a pollutant. However, ozone applied in aqueous solution and in a timely manner is expected to interact with plants differently than in the gaseous state, with a sufficiently high phytotoxic threshold that allows its incorporation in irrigation and spraying treatments in different crop species^11^. Unfortunately, literature concerning the effects of ozonated water on grapevine plants is scarce and almost exclusively dedicated to analysing its effect on microbial populations^3,4,12^, except a few recent studies describing its impact on grape and wine composition^12–16^.

The microvine is a convenient model plant for performing physiological studies in a semi-controlled environment. Carrying the *Vvigai1* mutation, microvines exhibit a continuous flowering, simultaneously displaying all the successive stages of fruit development on a single shoot^17^. This model has already facilitated transcriptomics approaches of the circadian cycle^18^, high-temperature stresses^19,20^, metabolomics works surveying glycosylated aroma precursors^21,22^, and several berry developmental studies^23–25^.

In this study, this model allowed us to characterise the early transcriptome changes triggered in grapevine leaves and berries at different ripening stages after *in planta* sprayings of ozonated water solutions.

## Results

### The balance in primary metabolites: an analytical tool to select RNA-Seq samples

**Figure 1:**
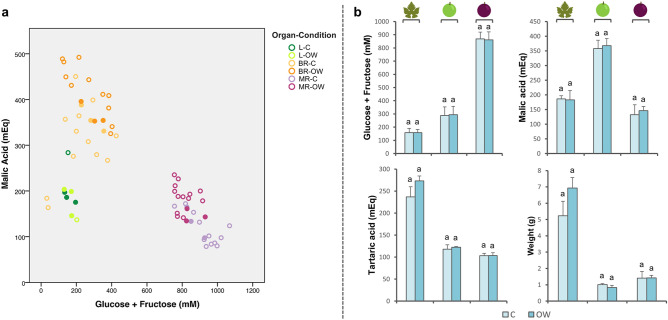
Sugar and acid content and organ fresh weight. (**a**) Malic acid (mEq) as a function of sugar (glucose + fructose, mM). Full coloured circles represent the individual berry or pairs of leaves selected for RNA-Seq; (**b**) Average concentrations in glucose + fructose (mM), malic acid (mEq), tartaric acid (mEq), and organ fresh weight (g) of the selected triplicates. Error bars represent the SD (n = 3). Selected samples showed no significant differences between conditions (C and OW) according to the independent samples t-test (*p* < 0.05). Figure was obtained with IBM SPSS Statistics 24 (https://www.ibm.com/products/spss-statistics).

At the beginning of ripening (BR), soft green berries were sampled while still in the lag phase with no visible anthocyanin accumulation in their skin. These berries just started to accumulate sugar while consuming malate (Fig. 1a). As expected, berries in the mid-ripening stage (MR) showed higher sugar concentrations (close to 1 M) and a lower amount of malic acid (Fig. 1a). Mature leaf samples (L) displayed a comparable amount of soluble sugars to BR, with a two-fold lower malate concentration, indicating strong differentiation between the source (leaves) and sink (berries) organs. Thanks to the measurements of sugars and acids, it was possible to gather synchronised samples^26^ for further RNA-Seq analysis with the aim to reduce biases in gene expression caused by the natural developmental asynchrony of grapevine berries and focus only on the early transcriptomic changes triggered by the ozonated water treatment. Indeed, biological triplicates were selected at the same sugar (glucose + fructose) concentrations for control (C) and ozonated water treatment (OW), namely 158 mM in L, 291 mM in BR, and 864 mM in MR (Fig. 1b). Malic and tartaric acids were 184 mEq and 255 mEq for L, 363 mEq and 120 mEq for BR, and 139 mEq and 103 mEq for MR (Fig. 1b), giving an average malate/tartrate ratio of 0.7, 3.0, and 1.3, respectively in L, BR and MR. No significant differences were found between conditions for sugar, acids and sample weight (Fig. 1b).

### Transcriptomic overview in leaf and ripening berry

**Figure 2:**
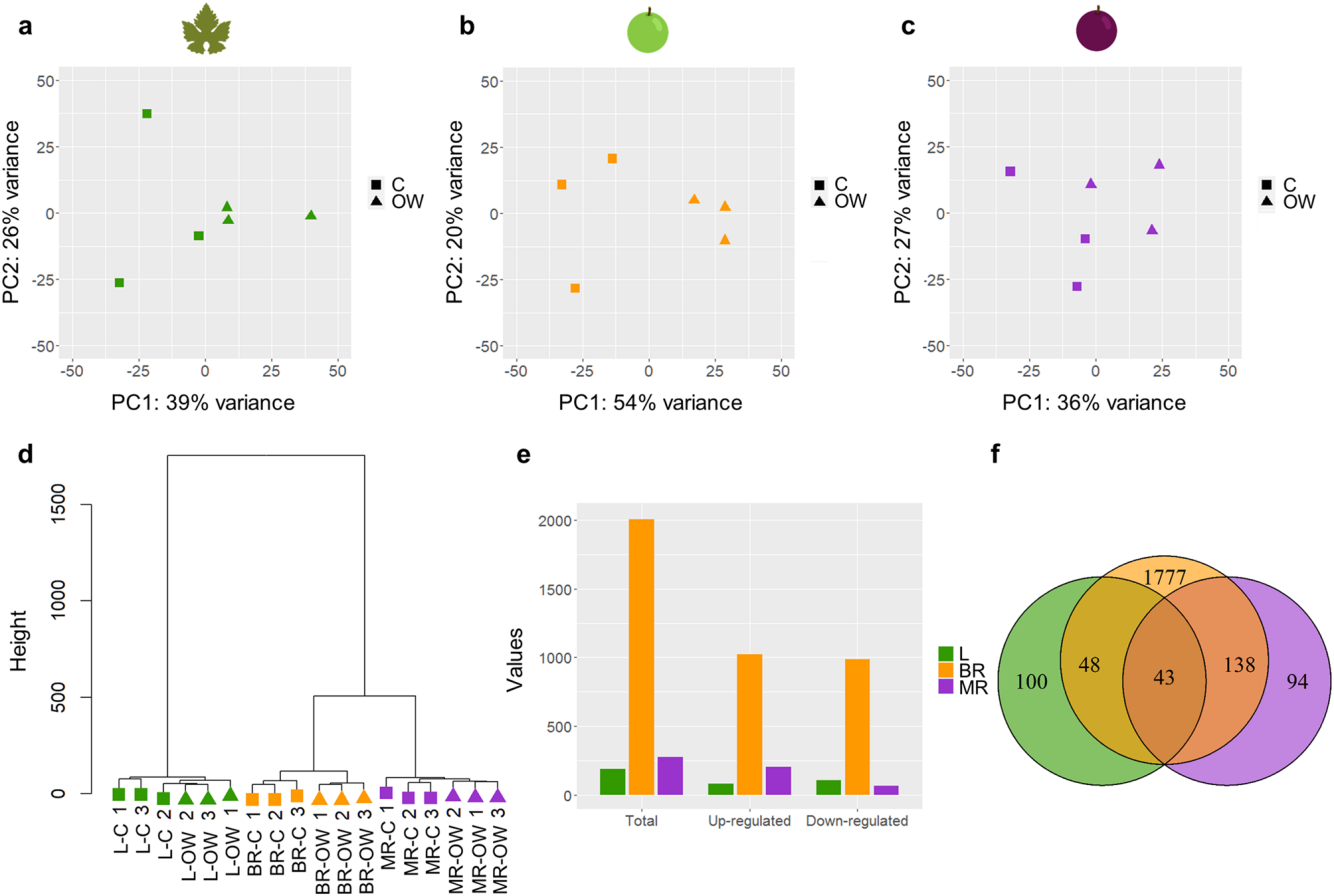
Transcriptomics overview. Principal component analysis of the transcriptomic samples in (**a**) leaves (L, green), (**b**) berries at the beginning of ripening (BR, orange), and (**c**) berries in mid-ripening (MR, purple); (**d**) sample dendrogram, with C and OW samples represented with a square and a triangle, respectively; (**e**) number of DEGs in L, BR and MR, and (**f**) commonly and uniquely modulated genes.

Principal component analyses were performed to visualise the global transcriptome trends (Fig. 2a–c). The first two principal components (PC1 + PC2) explained 65, 74, and 63% of the variance among samples in L, BR, and MR. C and OW samples were clearly resolved in BR, while the separation was less obvious in L and MR. The hierarchical clustering dendrogram showed the degree of similarity between the transcriptome profile of all the samples analysed (Fig. 2d). The most striking differences in the transcriptome were determined by the type of organ, i.e. leaf or berry, followed by the berry developmental stage. As before, the dendrogram showed that the C and OW BR samples grouped separately, while for L and MR the three OW replicates clustered conjointly, but one C sample was placed in a different branch than the other two.

Genes differentially expressed according to the ozone treatment were tested in the leaves and two berry developmental stages separately (Fig. 2e,f, and Supplementary Table S1). In L, the total number of DEGs was 191, with 84 up-regulated genes and 107 down-regulated. The most intense response to the treatment was observed in BR with 2006 DEGs, of which 1021 were up-regulated and 985 down-regulated. In MR, the treatment modulated the expression of 275 genes, with 207 up-regulated and 68 down-regulated. There were 43 commonly regulated DEGs between all the organs analysed, of which 40 up-regulated and 3 down-regulated. The DEGs commonly up-regulated in L, BR, and MR were mostly genes encoding heat shock proteins and chaperones, in addition to heat-stress transcription factor and galactinol synthase. Other genes among the up-regulated ones were a malate dehydrogenase, an argonaute protein, a RuBisCO large subunit-binding protein, several peptidyl-prolyl cis–trans isomerases, a calcyclin-binding protein, a NAD transporter and a putative SERF-like protein. Conversely, in the commonly down-regulated DEGs, we observed an auxin transporter-like protein and a pectin methylesterase (Supplementary Table S1).

**Figure 3:**
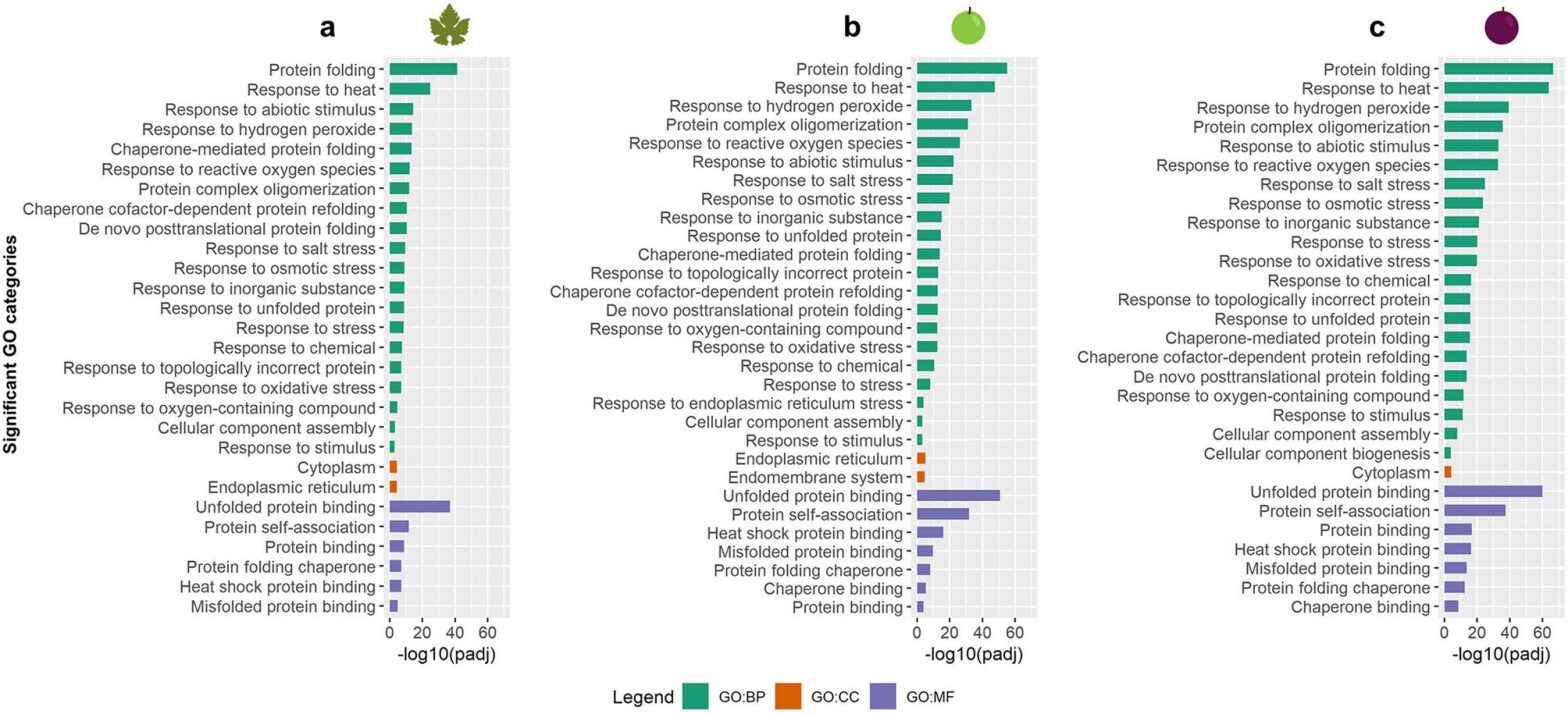
Significantly enriched GO categories in the up-regulated genes in (**a**) leaves, (**b**) berries at the beginning of ripening and (**c**) berries in mid-ripening, divided in biological process (BP, green), cellular component (CC, orange), and molecular function (MF, purple) expressed as − log_10_(adjusted p value).

The lists of up- and down-regulated genes were separately screened for significant enrichment (*p* < 0.001) in Gene Ontology (GO) categories in the Biological Process (BP), Cellular Component (CC), and Molecular Function (MF). The down-regulated genes fell in a limited number of enriched categories: for example, in L there were two CC categories such as *cell wall* and *external encapsulating structure*, in BR only *regulation of gene expression* for the BP, while no categories were enriched in MR. Instead, a higher number of categories and subcategories were detected in the up-regulated genes (Fig. 3). In common to the three organs, several categories reported enrichment for *protein folding* and related categories, and response to a plethora of stresses including *response to heat, response to hydrogen peroxide*, *response to reactive oxygen species*, and *response to oxidative stress* (Fig. 3).

### Up-regulation of heat-shock proteins and chaperones: a common response of leaves and berries

**Figure 4:**
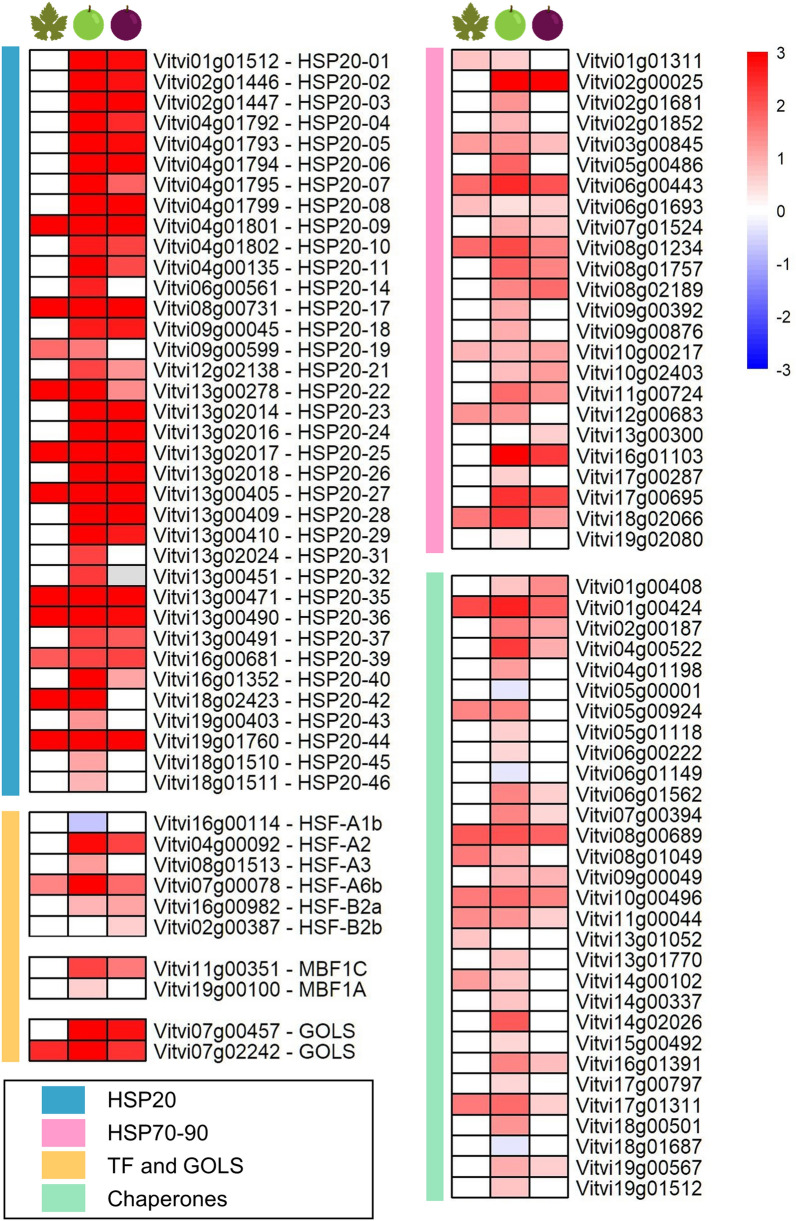
Heat-shock proteins, chaperones and related transcription factors modulated by the ozonated water treatment in leaves (left column), berries at the beginning of ripening (middle column) and berries in mid-ripening (right column) are represented in heatmaps as log_2_FC(OW/C). Red and blue colours indicate up- or down-regulation, respectively. The coloured sidebar on the left displays the class of genes.

To prioritise the genes whose expression changed the most after a 90-min exposure to ozone, each list of DEGs was ranked according to their respective absolute changes in expression between C and OW (Supplementary Table S1). The overall reaction in both leaves and berries was primarily to activate rather than repress physiological processes. The highest number of genes up-regulated by the OW treatment belonged to heat shock proteins (HSPs) and chaperones, with 11 small HSP or HSP20 genes upregulated in L, 36 in BR and 28 in MR (Fig. 4). Among the HSP20 recently identified in grapevine^27^, *VviHSP20-09*, *VviHSP20-17*, *VviHSP20-22*, *VviHSP20-25*, *VviHSP20-27*, *VviHSP20-35*, *VviHSP20-36*, *VviHSP20-39*, *VviHSP20-42*, and *VviHSP20-44* were commonly highly up-regulated. Other HSPs of higher molecular weight such as HSP70 and HSP90 were up-regulated as well, together with a series of chaperones, such as the DNAJ homolog, calnexin and calreticulin (Fig. 4).

Interestingly, diverse heat-stress transcription factors (HSFs) were also modulated by the treatment: *VviHSF-A6b* was up-regulated in L, BR, and MR. *VviHSF-A2* and *VviHSF-B2a* were up-regulated only in the berry (BR and MR). *VviHSF-A3* was up-regulated in BR, *VviHSF-B2b* in MR, and *VviHSF-A1b* was the only gene down-regulated in BR. Moreover, the transcription factors multiprotein-bridging factor 1c and 1a (*VviMBF1C, VviMBF1A*) were up-regulated, with a noticeably strong activation of the first one in the berries. Lastly, as part of the stress response, two galactinol synthases (*VviGOLS*) were up-regulated in L, BR, and MR (Fig. 4).

### Antioxidant homeostasis

**Figure 5:**
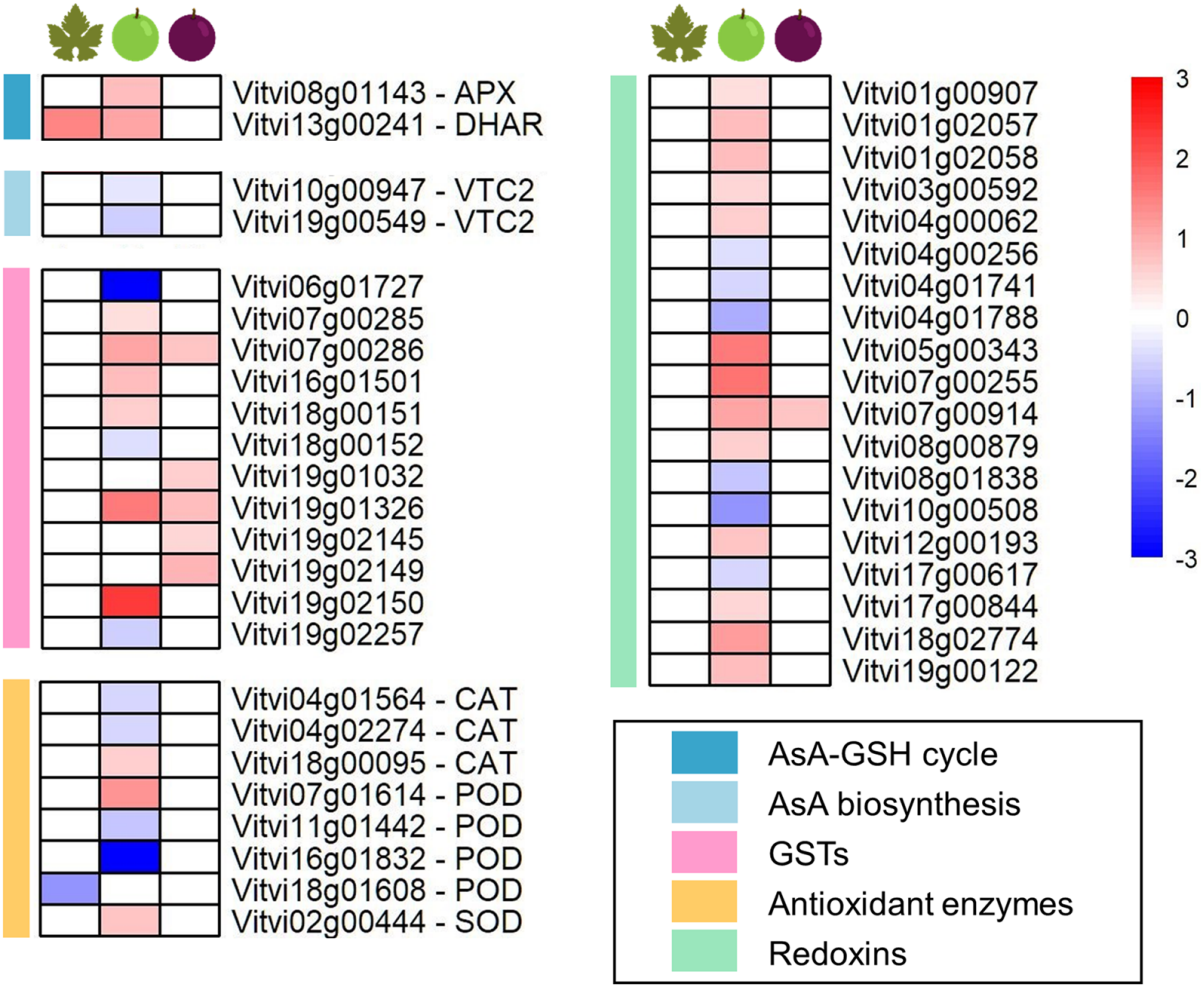
Oxidative stress-related genes modulated by the ozonated water treatment in leaves (left column), berries at the beginning of ripening (middle column) and berries in mid-ripening (right column) are represented in heatmaps as log_2_FC(OW/C). Red and blue colours indicate up- or down-regulation, respectively. The coloured sidebar on the left displays the class of genes.

Other categories of DEGs, identified mostly in BR, were related to the antioxidant homeostasis, which involves the scavenging of the reactive oxygen species (ROS). The ascorbate–glutathione cycle (AsA-GSH cycle) detoxifies ROS through the activity of ascorbate peroxidase (APX), monodehydroascorbate reductase (MDAR), dehydroascorbate reductase (DHAR), and glutathione reductase (GR), requiring a pool of ascorbate, glutathione and NADPH. Here, one APX (*Vitvi08g01143*) in BR and one DHAR (*Vitvi13g00241*) in L and BR were up-regulated (Fig. 5), indicating an enhanced turnover of the cycle under stress to scavenge O_3_-generated H_2_O_2_ into water. Paradoxically, two isogenes encoding *VTC2* (GDP-L-galactose phosphorylase), a regulatory step in AsA biosynthesis, were down-regulated in BR (Fig. 5).

Other antioxidant enzymes such as glutathione S-transferases (GSTs), catalases (CATs), peroxidases (PODs), superoxide dismutases (SODs) and redoxins (RXs) were modulated by the stress (Fig. 5). In particular, 9 GSTs were up-regulated (6 in BR and 5 in MR), while three were down-regulated in BR. Genes generally annotated as CAT (3), POD (4), chaperone for SOD (1), and RX (19) were modulated by OW in the BR berry with some genes induced and others repressed.

### Intense down-regulation of the cell wall-related genes and plasma membrane aquaporins

**Figure 6:**
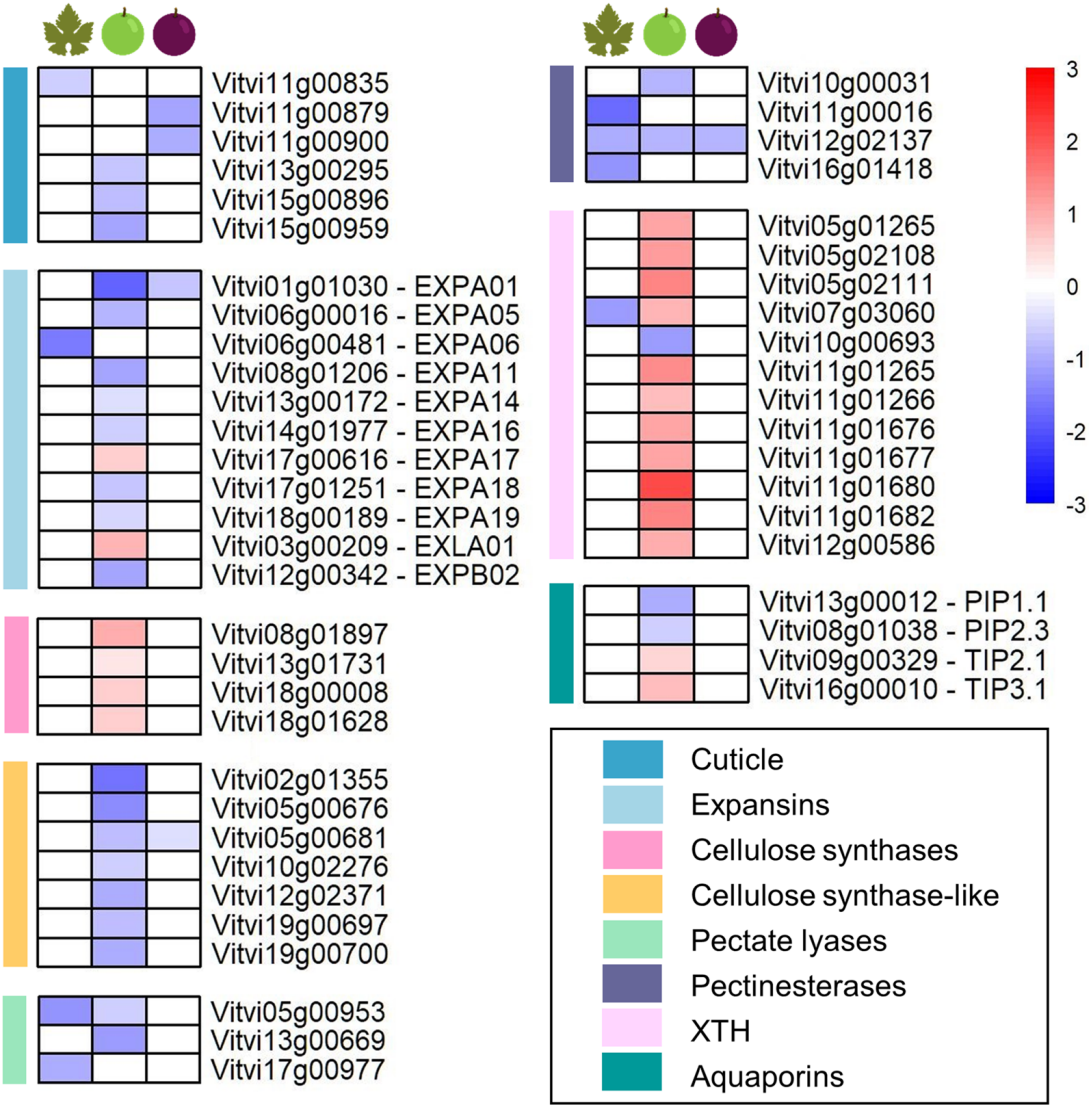
Cell wall and growth-related genes modulated by the ozonated water treatment in leaves (left column), berries at the beginning of ripening (middle column) and berries in mid-ripening (right column) are represented in heatmaps as log_2_FC(OW/C). Red and blue colours indicate up- or down-regulation, respectively. The coloured sidebar on the left displays the class of genes. XTH: xyloglucan endotransglucosylase/hydrolase.

Ozonated water sprayed all over the plant surface strongly impacted the cuticle and cell wall-related genes of leaves and berries with more emphasis on the BR berry (Fig. 6). Six cuticle genes were down-regulated together with ten expansins, among which the most highly repressed were *VviEXPA11*, *VviEXPA14*, *VviEXPA18*, and *VviEXPA19*. Four cellulose synthases and ten xyloglucan endotransglucosylase / hydrolase genes were up-regulated, while seven cellulose synthase-like were down-regulated together with three pectate lyases and other pectinesterases.

Interestingly, four aquaporins were modulated by OW at the beginning of ripening: *VviPIP1.1* and *VviPIP2.3* located on the plasma membrane were down-regulated; on the contrary *VviTIP2.1* and *VviTIP3.1*, whose localisation is the tonoplast, were up-regulated.

### Secondary metabolism is affected only at the beginning of ripening

**Figure 7:**
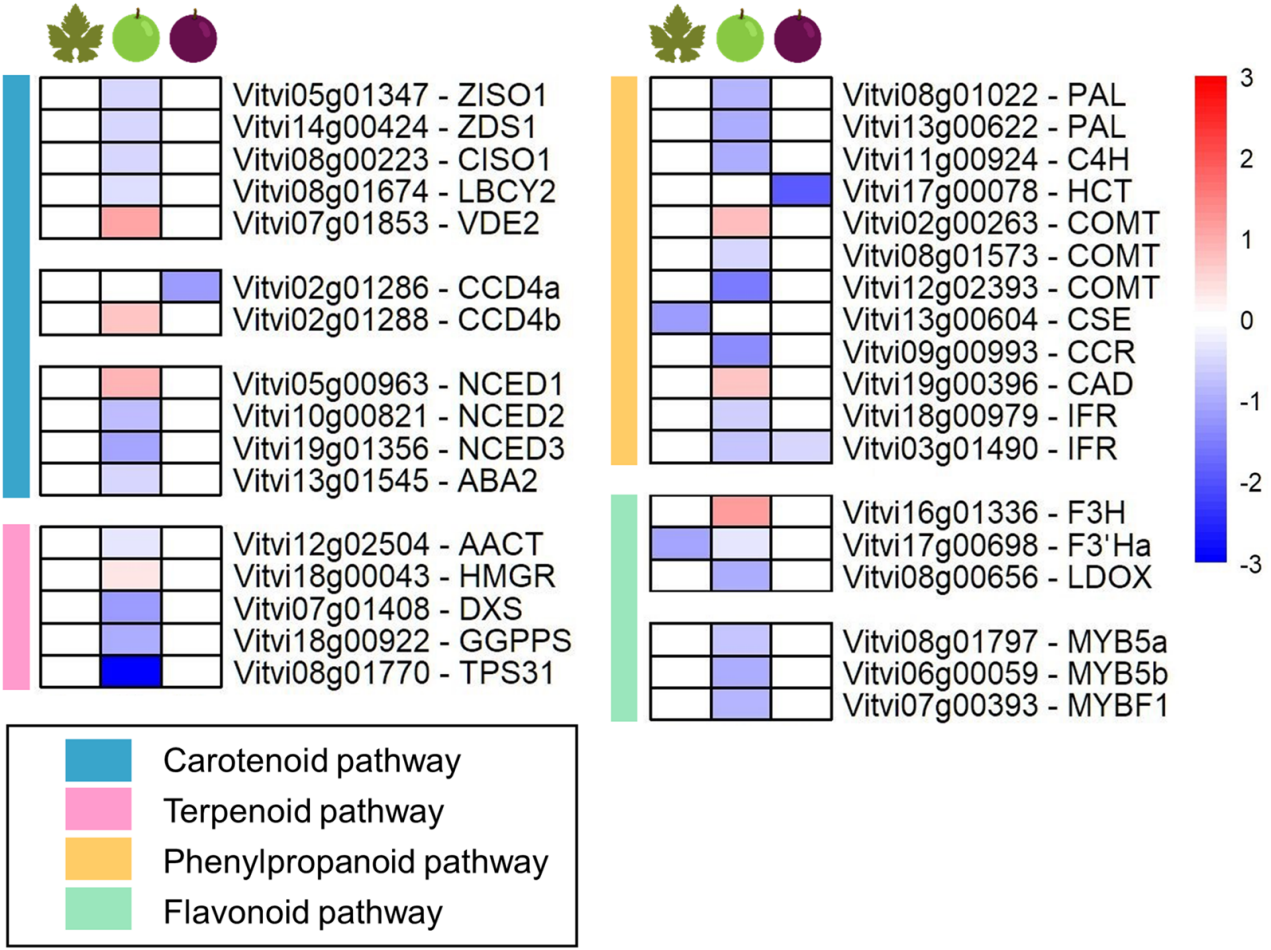
Secondary metabolites genes modulated by the ozonated water treatment in leaves (left column), berries at the beginning of ripening (middle column) and berries in mid-ripening (right column) are represented in heatmaps as log_2_FC(OW/C). Red and blue colours indicate up- or down-regulation, respectively. The coloured sidebar on the left displays the class of genes.

Antioxidant secondary metabolites like carotenoids, terpenoids and phenolic compounds can be synthesised in response to the stress. The expression level of several related genes was modulated by OW in BR berries, while no significant impact could be detected in L and MR (Fig. 7).

Genes involved in the early steps of carotenoid synthesis in grapevines such as a 15-cis-ζ-carotene isomerase (*VviZISO*1), a ζ-carotene desaturase (*VviZDS1*), a carotenoid isomerase (*VviCISO1*), and a lycopene β-cyclase (*VviLBCY2*) were down-regulated by OW in BR. Interestingly, a violaxanthin de-epoxidase (*VviVDE2*), involved in the violaxanthin and lutein epoxide (xanthophyll) cycles, was up-regulated. Carotenoids can be cleaved through carotenoid cleavage dioxygenases (CDD) to form volatile flavour and aroma compounds such as the C_13_-norisoprenoids (e.g. β-ionone and β-damascenone). The isoform *VviCCD4b* was up-regulated in BR, while *VviCCD4a* was down-regulated in MR. Lastly, neoxanthin and violaxanthin can also be cleaved by 9-cis-epoxycarotenoid dioxygenase (NCED) to form the hormone ABA; the three *VviNCED* were modulated with *VviNCED1* up-regulated, while *VviNCED2* and *VviNCED3* were down-regulated together with a xanthoxin dehydrogenase (*VviABA2*) in BR.

Plant terpenoids are synthesised via the cytosolic MVA and the plastidial MEP pathways. In the MVA, one acetyl-CoA acetyltransferase (*VviAACT*) was down-regulated in ozonated BR, while a hydroxymethylglutaryl-CoA reductase (*VviHMGR*) was up-regulated. In the MEP, the genes encoding a 1-deoxy-D-xylulose-5-phosphate synthase (*VviDXS*), a geranylgeranyl pyrophosphate synthase (*VviGGPPS*), and a terpene synthase (*VviTPS31)* were down-regulated in BR.

Several genes of the phenylpropanoid and flavonoid pathway were also differentially expressed by OW. In particular, two phenylalanine ammonia-lyases (*VviPAL*) and one trans-cinnamate-4-monooxygenase (*VviC4H*) were down-regulated in BR. In contrast, three caffeic acid 3-O-methyltransferases (*VviCOMT*) were differentially regulated with two genes down-regulated and one up-regulated. Other genes involved in the terminal steps of monolignol biosynthesis were also affected; namely, a cinnamoyl-CoA reductase (*VviCCR*) was down-regulated, while a cinnamyl alcohol dehydrogenase (*VviCAD*) up-regulated. In the same berries, two isoflavone reductases (*VviIFR*), implicated in the isoflavonoid phytoalexin branch pathway, were down-regulated. Lastly, the ozonation of MR berries only reduced the expression of a hydroxycinnamoyl-CoA: shikimate / quinate hydroxycinnamoyltransferase (*VviHCT*) and a *VviIFR*. Regarding the flavonoid pathway, a flavanone 3-hydroxylase (*VviF3H*), a flavonoid 3′-hydroxylase (*VviF3′Ha*) and a leucoanthocyanidin dioxygenase (*VviLDOX*) were modulated by the stress in BR: the first one up-regulated, while the last two down-regulated. A caffeoyl shikimate esterase (*VviCSE*) and the *VviF3′Ha* were the only DEGs down-regulated in L. Lastly, relevant transcription factors, such as *VviMYB5a*, *VviMYB5b*, and *VviMYBF1,* controlling different branches of the flavonoid pathway, were down-regulated in BR.

## Discussion

Although the major use of ozone in agriculture lies in its antifungal activities, as confirmed in grapevine^3,4,12^, there is still a lack of information on how ozone can affect grapevine physiology and grape composition. Besides, the role of ozone in preventing or controlling infections of *Plasmopara viticola* and *Erysiphe necator*, the agents responsible for downy and powdery mildews in grapevine, has not yet been confirmed. Previous field experiments reported versatile impacts of ozonated water sprayings on the composition in phenolic and terpenoid compounds of grapes and resulting wines^12–14,16^. Such discrepancies suggest that more studies in controlled conditions (e.g. with potted plants grown in greenhouses) are needed to describe the molecular and biochemical changes induced by O_3_ in grapevine organs. Using the microvine model, this study represents the first transcriptomics analysis exploring the responses triggered by ozonated water spraying on grapevine leaves and fruits.

BR berries appeared incredibly responsive to ozone exhibiting the highest number of DEGs. The intense transcriptomic reprogramming at the onset of ripening, largely documented in grapevine fruit^28,29^, has also been associated with ROS accumulation^30^, whose synthesis occurs most intensively during the night^18^. Due to the method of monitoring the development of the berries and their sampling, we can reasonably assume that BR berries were very close to the H_2_O_2_ and catalase peaks that were spotted in non-developmentally synchronised fruits^30^. The intense transcriptomic changes described here showed that endogenous ROS production previously reported at the onset of ripening is actually far from saturating in standard conditions with no stress. The observed response can also be explained by the greater variety of reactive species formed from aqueous O_3_, including the more potent oxidant and chain-propagating hydroxyl radical^6^, which can differ from the ones endogenously produced. In fact, the endogenous ripening related ROS production does not result in the cell wall and growth inhibition, as this production is suspected to occur just before or at the inception of the second fruit growth phase. Indeed, recent physiological and transcriptomic works evidenced that the less harmful hydrogen peroxide (H_2_O_2_) accelerated ripening in Kyoho variety^31,32^. The genes suggested by the authors to induce the early ripening were associated with the oxidative stress, photosynthesis, cell wall deacetylation and degradation. More studies are needed to decipher the possible role of ozonated water in grape ripening, knowing that H_2_O_2_ is only one of the ROS formed by the decomposition of ozone in aqueous solution^6^. Berry softening marks the onset of the massive import of sugars in grapevine. Surprisingly, *VviSWEET10*, which is implicated in the unloading of phloem sucrose inside the berry^25^, was up-regulated in BR (Supplementary Table S1) together with two TIPs, aquaporins of the vacuole. But the expression of *VviHT6*, the major sucrose transporter on the tonoplast, was not affected, leaving open the question of a possible enhancement of the ripening program under ozonated water. As ozone decomposition strongly depends on pH, its decay may be faster in the cell wall and cytoplasm than in the acidic vacuole of berries at the beginning of ripening^33^.

In our dataset based on developmentally synchronised berries, some cuticle related genes were down-regulated. The degradation of this protective barrier, which leads to greater penetration of ozone into the plant cells, has been reported in growing plants and postharvest fruits exposed to ozone^34^. Moreover, key expansins involved in the cellular expansion and growth^35^ were down-regulated with pectate lyases, pectinesterases and cellulose synthase-like genes indicating an immediate multifaceted effect unsettling the cell-wall dynamics, further exacerbated by the down-regulation of two plasma membrane aquaporins suggesting a limited water influx. Although all point toward a decrease in cell expansion, we did not observe any specific phenotypes on leaf or grape development (data not shown) in the following weeks after the treatment. This might indicate that ozone triggers only a transitory inhibition of cell wall remodelling and expansion. However, ozone has been shown to modify the composition and mechanical properties of grape skin cell walls^36^, affecting aroma and polyphenols extraction during winemaking^37^. The lower anthocyanin extractability observed after spraying ozonated water on grapevines^13,15^ may originate from the down-regulation of genes encoding pectin-degrading enzymes detected in ozonated berries.

The first coordinated response to the ozonated treatment was the induction of a plethora of HSPs and other chaperones. HSPs are involved in the cellular response to a diverse array of stresses, including oxidative^38^. They act mainly as molecular chaperones, participating in protein folding, assembly, translocation and degradation in many normal cellular processes and maintain proteins in their functional conformations under stress conditions, preventing their aggregation and denaturation, and assisting in protein refolding^39^. The induction of HSP transcripts in plants fumigated with ozone was first described in parsley^40^ and then confirmed in other plants such as *Arabidopsis thaliana* and *Medicago truncatula*^41,42^. Using proteomic approaches, the increased expression of these proteins under ozone stress was also detected in poplar, bean, maize and rice^43–45^. The induction of HSPs is under the tight control of an HSF network^46^, with significant players *VviHSF-A2* and *VviHSF-A6b* already reported intensified in grapevine under stress^19, 20,47^, often together with *VviGOLS*^48^. Moreover, transgenic *Arabidopsis thaliana* plants constitutively expressing the transcriptional coactivator *AtMBF1c* showed enhanced tolerance to environmental stresses^49^. Here these genes were strongly up-regulated, possibly cross-regulating several plant response mechanisms to various stresses.

Plants submitted to abiotic or biotic stresses typically produce ROS, triggering oxidative stress^50,51^. AsA is the most abundant antioxidant in plant cells, found in all subcellular compartments, including the apoplast, and therefore representing the first line of defence against ozone^52^. AsA can directly scavenge ozone and different ROS^53^ and, along with glutathione in the AsA-GSH cycle, is the primary H_2_O_2_ reducing substrate operating in cytosol, chloroplasts and mitochondria of plant cells^54^. It has been shown that the antioxidant response to the stress is genotype-dependent, with grape varieties such as Touriga Nacional able to boost the cell redox‐buffering capacity with the existing AsA and GSH pools, while other varieties, like Trincadeira, need to synthesise both metabolites because of its incapacity to keep the cellular redox state at working levels^55^. Therefore, it is not surprising that *VviVTC2*, the central regulator of the AsA biosynthetic pathway^56^, was down-regulated in BR, indicating a non-need for resynthesis but a buffering capacity of the microvine coping with oxidative stress. Similarly to our results, *OsVTC2* was down-regulated in ozone-exposed rice, attributing the changes in total and reduced AsA concentration to AsA turnover rather than biosynthesis, with a parallel increase of *OsAPX*, *OsDHAR*, and *OsGR*^57^. Also in our dataset, *VviAPX* and *VviDHAR* were up-regulated under ozone. Elevated expression of these two genes in response to ozone has already been detected in *Arabidopsis thaliana*^58,59^, and DHAR-overexpressing plants have shown increased tolerance to ozone by incrementing foliar AsA level^60^. In grapevine, AsA is also a precursor for the synthesis of both tartaric and oxalic acids. The down-regulation of *VviVTC2* in BR berry under ozone stress could indicate a switch from the Smirnoff-Wheeler (SW) pathway to the alternative AsA biosynthetic pathway, knowing that the first one supports AsA biosynthesis in immature berries, while the alternative synthesises AsA from a methyl derivative of D-galacturonic acid released during pectin degradation as fruits ripen^61^. Given that GDP-D-mannose and GDP-L-galactose, intermediates of the SW pathway, are also precursors of the non-cellulosic components of the plant cell wall^62^, we can speculate that the inhibition of enzymes involved in cell wall synthesis and growth would lead to AsA sparing and in turn to reduced AsA synthesis, materialised through the down-regulation of *VviVTC2*.

Other critical antioxidant enzymes such as CAT, POD, SOD, RX, and GST were modulated by the stress indicating an intense redox homeostasis activity to prevent damage from ozone and its by-products^50^. In particular, the treatment induced the expression of six out of nine GSTs detected in BR berries. This elicitor effect was also observed in MR berries, confirming previous results in ozone-exposed *Arabidopsis* and rice seedlings^41,45,63^. Thiols such as GSH are versatile targets for most oxidants, including ozone^64^, so we hypothesise that GST transcript levels increased in order to counterbalance reduced substrate availability. In addition, this alleged reduced availability of GSH may have been enhanced in the BR berry by *VviDHAR* up-regulation. GSTs are also necessary for the transport of anthocyanins from the cytosol to the vacuole. Consequently, a strong correlation between these proteins and anthocyanin accumulation has been found in *V. vinifera*^65^, indicating a possible involvement in the increased phenolic content under ozonated water treatments.

Although many secondary metabolites are important antioxidants whose synthesis is typically induced in plants as a defence mechanism against ozone^8, 9^, in the early transcriptional response to the ozonated water application their pathways were generally unaffected in leaves and mid-ripening berries, with some genes down-regulated in berries starting to ripen.

Carotenoids contribute to light harvesting and protect the photosynthetic membrane against photo-oxidative damage, not only by quenching the triplet states of chlorophyll but also by scavenging ROS^66^. A possible impairment of carotenoid synthesis through the down-regulation of *VviZISO1*, *VviZDS1*, *VviCISO1* and *VviLBCY2* in the early ripening berry seems counter-intuitive, however, similar observations were made in different rice genotypes^57^. The regeneration of carotenes and xanthophylls from their oxidised radicals relies on AsA^53^ and, in addition, the violaxanthin de-epoxidase enzyme requires AsA as a cofactor^67^. Here, the higher expression of *VviVDE2* in the ozonated BR berry indicates an activation of the de-epoxidation in the xanthophyll cycles, which protects against ROS-generating stresses^68^. This mechanism is expected to be also activated in ozone-treated leaves as they often undergo a reduction in photosynthetic rates and need to dissipate the excess excitation energy absorbed by the antennae^10^. However, here, no sign of photosynthetic apparatus damage was observed in leaves. The activation of the xanthophyll cycles in BR berry may respond to the zeaxanthin and lutein roles in ROS scavenging and preventing membrane lipid peroxidation^69,70^.

Terpenoids have been shown to improve the ability of plants to cope with internal oxidative changes^71^, reduce ozone damage and quench ozone and ROS^9^. However, ozone has been shown to stimulate and reduce the biosynthesis and emission of these volatiles depending on the severity and duration of the exposure and the plant species sensitivity^72^. Here, the overall down-regulation of the genes involved in their synthesis in the treated BR berries, such as *VviDXS* and *VviTPS31*, key determinants in the production of monoterpenes in grapevines^73,74^, and *VviGGPPS,* the precursor of diterpenes and carotenoids, contrasts with the higher terpenoid content found in berries from Bobal and Vermentino grapevines subjected to ozonated water treatments^12, 13^. Nevertheless, this increase was detected in berries at the end of the ripening period and not after each ozone exposure, and was much less pronounced when the treatment was applied at the onset of veraison^13^. It should be noted however, that the transcriptional profiles reported in the current study represent an early response, while longer-term effects on expression (e.g. at the time of harvest) could vary. In this line, an immediate depression of isoprene emission was reported in *Quercus pubescens* leaves exposed to ozone, attributed to a temporary inhibition of photosynthesis, but a subsequent fast recovery and even stimulation 12 days after fumigation^75^.

Plants exposed to ozone often respond with increased transcription and activities of enzymes involved in the phenylpropanoid, lignin and flavonoid pathways because of their barrier and antioxidant roles^10,76^. However, this response may not be immediate: for example, the induction of genes involved in the flavonoid synthesis in *Arabidopsis* was part of the later response to two days of ozone exposure, with chalcone synthase, dihydroflavonol reductase and leucoanthocyanidin dioxygenase being the most responsive^41^. In *Melissa officinalis* L., an ozone treatment (5 h) initially impaired PAL activity, the first enzyme in the general phenylpropanoid pathway, followed by a subsequent increase 7 h after the end of the exposure^77^. Similarly, our results showed that the early response to the ozonated water treatment, mainly in BR berries, consisted of an overall down-regulation of several genes involved in these pathways. Whether these genes are reactivated later is presently unknown.

Plants are sessile organisms that produce metabolites as an adaptive strategy to cope with challenging and changing environments^78^. Secondary metabolic routes are highly demanding for energy and carbon compounds, including the metabolites synthesis, their transcriptional regulation, and transport in subcellular compartments^79^. On the urgency to respond to the stress in the short-time, grapevine vegetative and reproductive organs apparently prefer to allocate carbon and energy to immediate defence response (HSPs, chaperones, AsA-GSH cycle). We can speculate that multiple treatments and/or a longer span between ozone exposure and sampling could lead to adaptation mechanisms triggering cascades of signal networks ending with the synthesis of stress-related genes and secondary metabolites accumulation, as often observed in grapes at harvest. This study is an original contribution performed with a perennial fruit crop. The goal was to characterise the first responses of both vegetative organs and fleshy fruits to ozonated water treatments (Supplementary Figure S1). Therefore, further studies will be needed to get a comprehensive understanding of the long-term effects on plant physiology and especially on fruit composition. Based on this first study and previous experiences^17^, we propose the microvine as a relevant perennial fleshy fruit model to perform such investigations.

## Methods

### Plant material

Two-year-old ML1 microvines were grown in 3 L pots under semi-controlled conditions in a greenhouse (Montpellier SupAgro- INRAe campus, France) with day/night temperature 25/15 °C, 1 kPa of VPD, and 12 h photoperiod. Microvine ML1, which was obtained by L. Torregrosa^17^, complies with relevant institutional, national, and international guidelines and legislation. Plants were managed for eight months to display all fruit developmental stages from flowering to ripe stages^17^. Plants were maintained at full ETP (*EvapoTranspiration Potential*), thus avoiding water stress issues, and no fungicide sprayings were performed. At the beginning of the experiment, the developmental stage of single green berries was checked by visual inspection and firmness assessment^23^, in order to detect the first softening signs as the onset of sugar storage. Single berry growth was weekly monitored by image analysis of clusters taken 30 internodes below the apex, with a Lumix FZ100 camera (Panasonic).

### Ozonated water treatment and sampling

**Figure 8:**
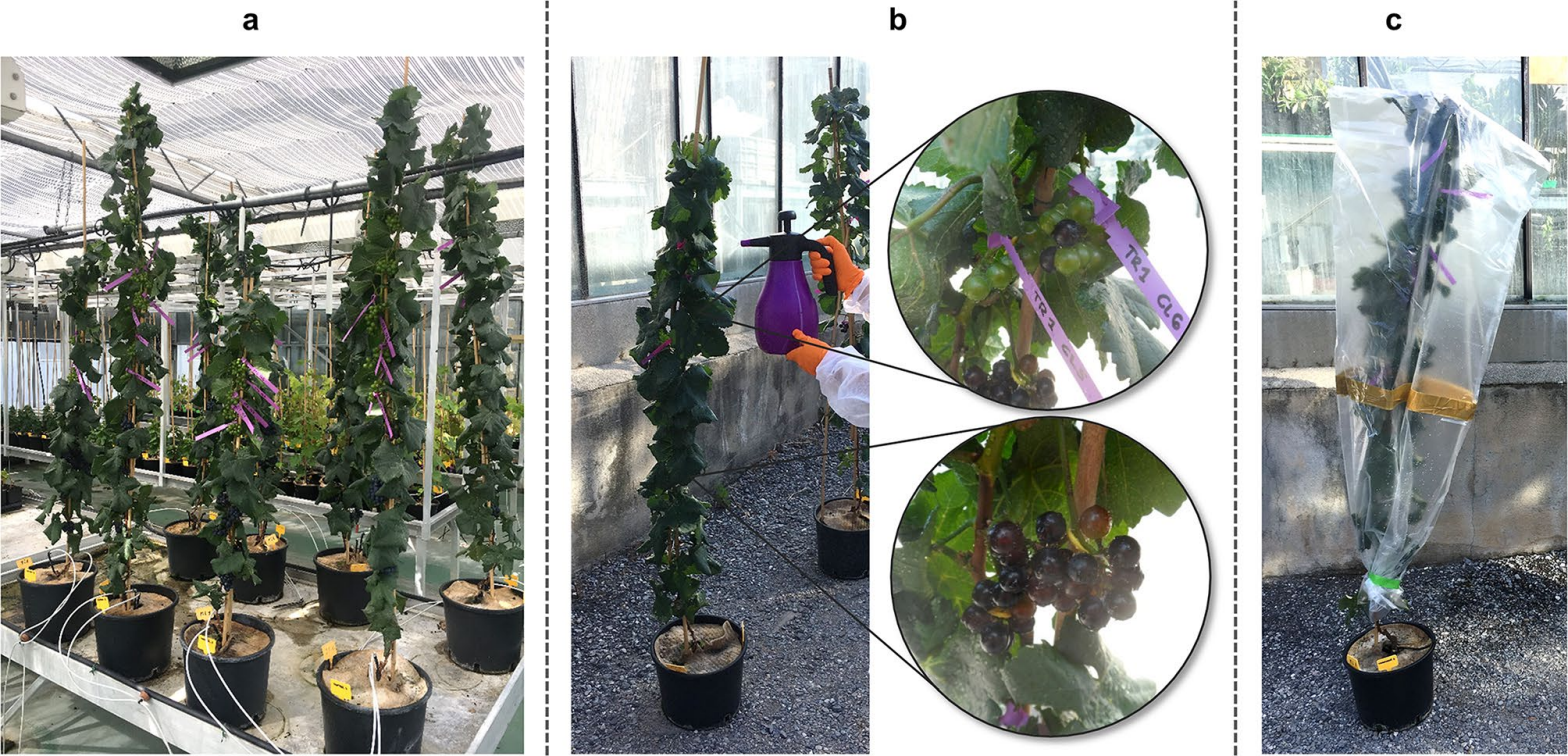
Experimental setup. (**a**) Microvine plants in the greenhouse; (**b**) ozonated water spraying treatment on plants showing different berry developmental stages; (**c**) microvine covered with a plastic bag immediately after the treatment.

Before treatment, plants were randomly divided into two groups: four plants for the control (C) and four plants for the ozonated water treatment (OW) (Fig. 8a). To have ventilation representative of field conditions and prevent off-target ozone diffusion, plants were brought outside the greenhouse for the entire duration of the experiment (9 am–12 pm), all C and OW plants being managed in the same environmental conditions. To avoid additional stress (e.g. excessive temperature or light exposure), the experiment was done in the morning at a time when the temperature was similar to that of the greenhouse (25 °C) and the plants were kept in the shade. In addition, the plants were maintained outside for about one hour prior to treatment to allow them to adapt to the outdoor environment. Ozonated water was prepared extemporaneously using an ozone generator (Cosemar Ozono S.L., Spain) connected to a sprayer containing Milli-Q water at a temperature of 15 °C and a conductivity of 18.2 MΩ/cm. A redox meter (PCE-228-R, PCE Ibérica S.L., Spain) was used to continuously measure in millivolts (mV) the oxidation–reduction potential (ORP) of the aqueous solution. One hundred fifty mL of ozonated water (once its ORP reached 1000 mV) was sprayed on the entire surface of each OW plant (Fig. 8b). The four C vines were sprayed with the same amount of Milli-Q water used for the treatment. Immediately after the spraying, plants were enclosed in plastic bags to prevent drift and avoid too rapid ozonated water evaporation (Fig. 8c), in an attempt to mimic field conditions where the spraying is usually carried out early in the morning, at low wind. Ninety minutes after the start of the treatment, 15 single individual green berries at the beginning of ripening (BR) (+ 3 days after softening), 15 single individual berries in the mid-ripening stage (MR) (+ 18 days after softening), and two adult leaves per plant (L) located between the 30th and 40th nodes were sampled for both C and OW. Single berry samples (pericarp and seeds) and leaves were wrapped separately in aluminium foils and immediately frozen in liquid N_2_. Each sample was weighed and ground into liquid N_2_ using a ball mill (Retsch, Germany). The resulting powder was stored at − 80 °C, and used for primary metabolites and RNA analyses.

### Primary metabolites analysis

Sugars and acids were analysed by high-performance liquid chromatography (HPLC), according to Rienth et al.^20^. Briefly, 100 mg of leaf or berry frozen powder was 5 × diluted in HCl 0.25 N and left overnight at room temperature after shaking. Samples were then centrifuged at 15,000 g for 10 min, and a supernatant aliquot was diluted 10 × with a solution of H_2_SO_4_ 5 mM containing 600 µM acetic acid as internal standard, before injection into the HPLC system. The statistical analysis of the data was performed with SPSS statistics software (version 23.0 for Windows, Chicago, IL, USA). The mean values of the selected samples were compared using the independent samples t-test, and the differences were considered statistically significant when the p-value < 0.05.

### RNA extraction and sequencing

Three samples per treatment (C and OW) and organ (L, BR and MR) were selected for individual RNA extraction and library preparation as described in Rienth et al.^80^. Samples were sequenced on an Illumina HiSeq3000 in paired-end mode, 2 × 150 bp reads, at the Genotoul platform of INRAe-Toulouse (France).

### Data analysis

Raw reads were trimmed for quality and length with Trimmomatic, version 0.38^81^. Reads were aligned against the reference grapevine genome PN40024 12X2^82^, using the software Hisat2, version 2.1.0^83^ with standard parameters, yielding an average of 25.3 M sequence per sample (Supplementary Table S2). Aligned reads were counted using the VCost.v3 annotation with HTSeq-count (version 0.9.1)^84^, in union mode, mRNA type, nonunique all, and stranded options. Only genes with RPKM > 1 were kept for further analysis (Supplementary Table S3). Differentially expressed genes (DEGs) (FDR < 0.05) were screened with the R package DeSeq2^85^. Overrepresented gene categories were identified with the gProfiler web-server (version 101_eg48_p14_baf17f0) with a significance threshold of 0.001. PCA and dendogram figures were drawn with RStudio (package ggplot2). Heat map figures were drawn with RStudio (package pheatmap v1.0.12).

## Supplementary information

Supplementary Figure S1

Supplementary Table S1

Supplementary Table S2

Supplementary Table S3

## Data Availability

Raw transcriptomics reads have been deposited in NCBI Sequence Read Archive (http://www.ncbi.nlm.nih.gov/sra). The BioProject is PRJNA678610.
