# Supplementary figures and images for: The application of ozonated water rearranges the Vitis vinifera L. leaf and berry transcriptomes eliciting defence and antioxidant responses
Source: Sci Rep. 2021 Apr 14;11:8114. doi: 10.1038/s41598-021-87542-y (PMC8046768; doi:10.1038/s41598-021-87542-y)

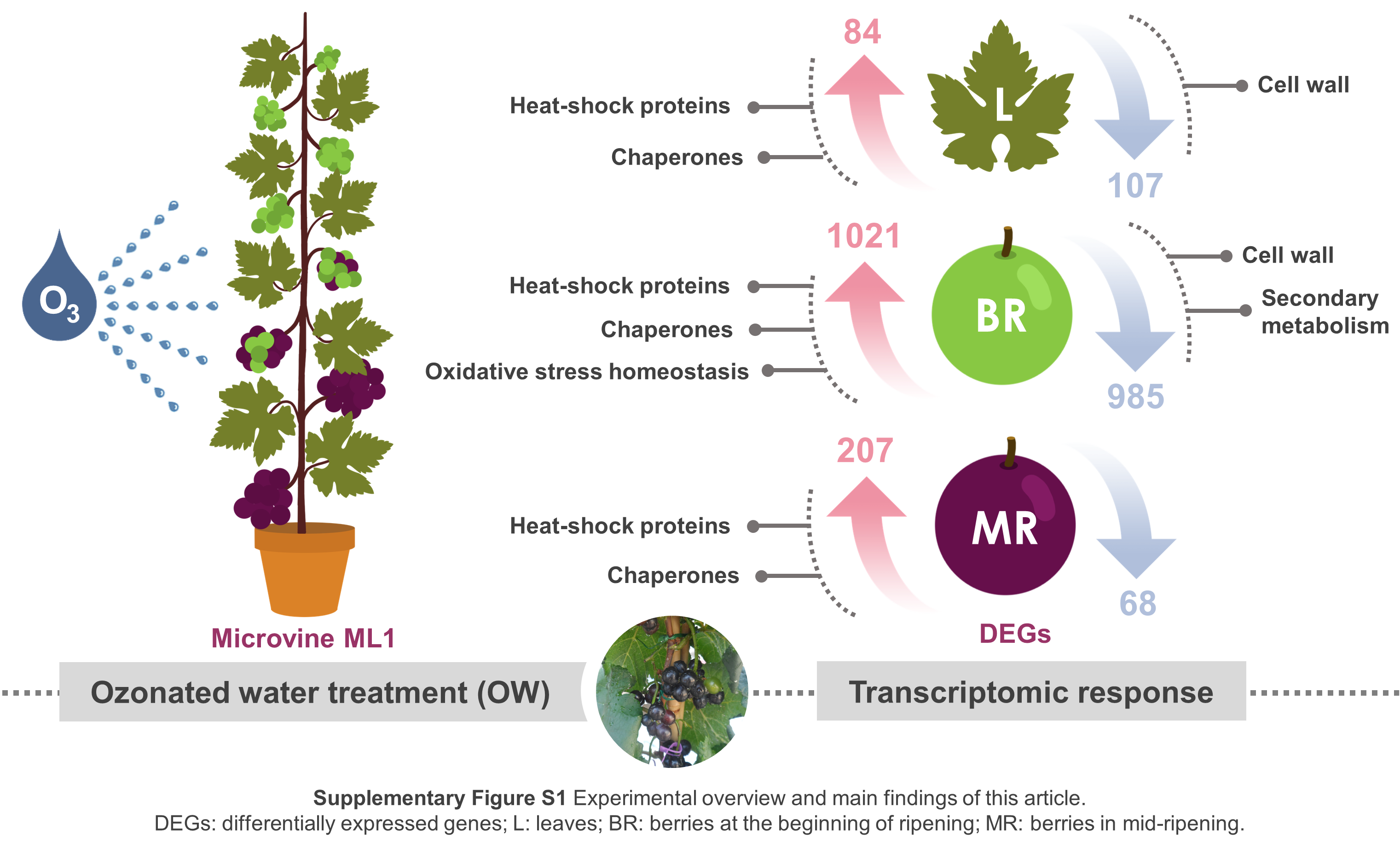

Supplement: Supplementary file 1 — Supplementary Figure S1 [file 41598_2021_87542_MOESM1_ESM.tif]
